# Infectious complications of immune checkpoint inhibitors in solid organ malignancies

**DOI:** 10.1002/cam4.4393

**Published:** 2021-12-07

**Authors:** Justine Abella Ross, Kellie Komoda, Sumanta Pal, Jana Dickter, Ravi Salgia, Sanjeet Dadwal

**Affiliations:** ^1^ Department of Pharmacy City of Hope National Medical Center Duarte California USA; ^2^ Department of Medical Oncology City of Hope National Medical Center Duarte California USA; ^3^ Division of Infectious Diseases Department of Medicine City of Hope National Medical Center Duarte California USA

**Keywords:** checkpoint blockade, immunotherapy, infection, melanoma, nonsmall‐cell lung cancer, renal cell carcinoma

## Abstract

**Background:**

Immune checkpoint inhibitors (ICIs) are targeted cancer therapies regarded to have less toxicity than chemotherapy. Immune‐related adverse events (irAEs) of ICIs are well described in the literature but limited data exist on their infectious complications. The objective is to describe the spectrum and risk factors for developing serious infections in patients receiving ICIs.

**Methods:**

Retrospective review of patients with melanoma, renal cell carcinoma, or nonsmall‐cell lung cancer on nivolumab, pembrolizumab, and/or ipilimumab from January 1, 2017 to November 30, 2017. Exclusion: receipt of less than two ICI doses or history of other malignancy. Characteristics: age, sex, prior chemotherapy, steroid use, and temozolomide or infliximab use. Data identified from microbiology, radiography, serology, or physician note documentation. Serious infection is defined as infections requiring hospitalization and/or IV antibiotics from initiation of ICI until the end of the study period.

**Results:**

One hundred and eleven pts received ICIs. Suspected or confirmed bacterial infections occurred in 24% (27/111) with 8% (9/111) confirmed bacterial cultures. The overall serious infection rate was 14% (16/111) with 25% (4/16) confirmed bacterial cultures. Suspected or confirmed infection sites: genitourinary 20% (22/111), pneumonia 5% (7/111), skin/soft tissue 7% (8/111). Noninfectious pneumonitis (NIP) occurred in 5% (5/111). No association regarding the risk of infection between the type of malignancy and ICI used. Steroid use was the only risk factor significantly associated with serious infection: 12/16 (75%) on steroids versus 27/95 (28.4%) without steroid use (*p* = 0.0003).

**Conclusion:**

The rate of serious infection with ICI was higher in our study compared with previous reports of pts treated with melanoma. Infectious complications are encountered with ICIs and correlate with steroid use.

## Graphical abstract

The objective of our study is to describe the spectrum and risk factors for developing serious infections in patients receiving immunotherapy. The overall serious infection rate was 14% with bacterial infections accounting for 37% of serious infections. Serious infections were associated with concomitant corticosteroid use.

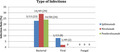

## 1 BACKGROUND

Immune checkpoint inhibitors (ICIs) including programmed cell death 1 (PD‐1) inhibitors and cytotoxic T‐lymphocyte‐associated antigen 4 (CTLA‐4) inhibitors are novel targeted cancer therapies regarded as having less toxicity than chemotherapy agents. ICIs permit immune stimulation by allowing unrestricted immune activation targeted toward tumor cells.^1^ CTLA‐4 is an inhibitory component on T cells, which inhibits a stimulatory signal necessary for T cell priming.^2^ Inhibition of CTLA‐4 allows T cell activation.^2^ PD‐1 is a receptor expressed on activated T cells, which interacts with PD‐L1 to reduce autoimmunity.^2^ When this interaction is inhibited, the immune response against tumor cells is enhanced.^2^ Immune‐related adverse effects (iRAEs) occur in response to augmentation of the immune system and may develop at different sites other than cancer.^3^ The type of immune‐checkpoint inhibitor regimen may also increase the risk of iRAEs. A recent study by Chang and colleagues report that pembrolizumab and nivolumab may have a lower risk of any irAEs compared with ipilimumab or nivolumab and ipilimumab combination therapy.^4^ Dermal iRAEs are most commonly occurring in 20%–50% of patients and present with rash or pruritus within the first two cycles.^3^ Gastrointestinal iRAEs are also common but occur approximately 5–10 weeks after initiation of immune checkpoint inhibitor therapy. Lung iRAEs present as noninfectious pneumonitis (NIP) and occur less commonly with an incidence rate of 5% and the onset of 9–20 months after initiation of therapy.^5^ Other less common irAE include endocrinopathy, neurologic complications, and cardiovascular toxicity. Treatment of iRAEs includes supportive care along with corticosteroids. Patients with refractory iRAEs may require additional immunosuppressive agents such as infliximab, cyclophosphamide, and mycophenolate mofetil.^6^

Clinical trials describe lung involvement with nivolumab, ipilimumab, and pembrolizumab with a reported rate ranging from ≤1% to 13%.^7^ Incidence rate of genitourinary tract infections ranges between 15% and 19% and upper respiratory tract infections 11%–44% in clinical trials.^7^ Pembrolizumab had infrequent incidences of herpes zoster (≥1%) and urosepsis (≥2%).^7^ Few studies describe the spectrum of infections associated with ICIs. Limited preclinical and clinical data suggest risk factors for infection include the immune modulation and use of immunosuppressive agents to treat irAE. A study in melanoma patients found that serious infections occurred in 7.3% of melanoma patients and the risk factors associated with infection were corticosteroid and infliximab use.^1^ Two recent studies have reported infection rates between 18 and 19% in patients receiving ICIs. In both studies, corticosteroid use was not associated with increased risk of infection; however, one report did identify a history of diabetes mellitus as a risk factor for infection.^2^, ^3^ The purpose of this study is to describe the spectrum of infections among patients with melanoma, renal cell carcinoma, or nonsmall‐cell lung cancer who were treated with ICIs; ipilimumab, nivolumab, and/or pembrolizumab. The secondary objective is to identify risk factors for serious infections with the use of the above‐mentioned ICI’s.

## 2 METHODS

### 2.1 Study design

This single‐center, retrospective review was approved by the City of Hope National Medical Center Investigational Review Board. All patients diagnosed with melanoma, renal cell carcinoma, or nonsmall‐cell lung cancer, receiving nivolumab, pembrolizumab, and/or ipilimumab from January 1 to November 30, 2017, were included. Exclusion criteria consisted of patients who received less than two doses of ICI or on nivolumab, pembrolizumab, or ipilimumab for diagnosis other than melanoma, renal cell carcinoma, or nonsmall‐cell lung cancer.

### 2.2 Data collection

Baseline characteristics collected included age, sex, prior chemotherapy, steroid use, and temozolomide or infliximab use. Infection type, site of infection, and organism were identified from microbiology and other laboratory data such as serology, imaging, and physician progress notes. Diagnostic testing typically involves respiratory sample (sputum or bronchoalveolar fluid) stain and culture for bacteria, fungi, and mycobacteria, BioFire™ respiratory polymerase chain reaction (PCR) panel, aspergillus galactomannan antigen (serum or bronchoalveolar lavage [BAL]), serum beta‐B glucan, mucor PCR *Pneumocystis jiroveci* direct fluorescent antibody, and PCR (both from BAL) are performed as needed in patients at risk for invasive mold infections or *Pneumocystis jiroveci* per European Organization for Research and Treatment of Cancer and the Mycoses Study Group Education and Research Consortium (EORTC/MSGERC) criteria in those who had pneumonia.^8^ Patients suspected to have infection also had blood culture, and urine culture if symptomatic. Radiographic data in conjunction with clinical data were reviewed by a physician to differentiate between pneumonia and NIP. Infectious pneumonia was defined as any symptoms of fever, productive cough, dyspnea, and hypoxemia at the time of imaging accompanied by radiographic abnormalities ranging from bilateral ground‐glass opacities, lobar consolidations, nodular infiltrate with or without cavitation, or multifocal infiltrates. NIP was defined as diffuse infiltrates in both lung fields deemed to be related to ICI and workup for infection was unrevealing. Serious infections were defined as suspected or confirmed infections requiring hospitalization and/or intravenous antibiotics. The uncomplicated group was defined as nonhospitalized patients who were suspected or confirmed to have infections and prescribed oral antibiotics in an outpatient setting or patients without any infection during the study period. Potential risk factors for infection include steroid use (≥10 mg of prednisone or steroid equivalent for at least 10 days from initiation of ICI), number of ICI doses, duration of therapy, history and concurrent use of chemotherapy, combination ICI, temozolomide, and infliximab use.

### 2.3 Statistical analysis

Analysis of variance (ANOVA) was used to analyze continuous data such as age, number of doses, and duration of therapy of ICIs. Chi‐squared test was used for categorical data such as sex, prior chemotherapy, and steroid use. A *p* value of <0.05 was considered significant.

## 3 RESULTS

### 3.1 Patient characteristics

Hundred and eleven patients receiving ICIs were identified, 7 of which received combination ICI. Thirteen patients received ipilimumab, 49 received nivolumab, and 58 received pembrolizumab for melanoma, nonsmall‐cell lung cancer, and renal cell carcinoma which also includes patients receiving more than one ICI at different times during the study period (Table 1). The mean age was 65.6 years (range 23–88) and the majority were males (63%). Ipilimumab had predominantly melanoma patients (69%) compared with nonsmall‐cell lung cancer (15%) and renal cell carcinoma (15%). Most patients treated with nivolumab were diagnosed with renal cell carcinoma (59%) compared with nonsmall‐cell lung cancer (24%) and melanoma (16%). Pembrolizumab was mostly used for nonsmall‐cell lung cancer (60%) compared with melanoma (28%) and renal cell carcinoma (12%). Six patients received ipilimumab in combination with nivolumab for melanoma (3/6), nonsmall‐cell lung cancer (2/6), and renal cell carcinoma (1/6). Ipilimumab in combination with pembrolizumab was used in one patient with melanoma. Fifty‐six percent received prior chemotherapy and 25% received concomitant chemotherapy with an ICI. Thirty‐two percent of patients received steroids for management of iRAEs, whereas none received temozolomide or infliximab. The mean number of ICI doses was 6.1 and the mean duration of therapy was 120 days.

**TABLE 1:** Patient characteristics

Characteristics	Total (*n* = 111)^a^	Ipilimumab (*n* = 13)	Nivolumab (*n* = 49)	Pembrolizumab (*n* = 58)	*p* value
Age					
Mean (Range)	65.6 (23–88)	50.1 (23–71)	65.4 (39–85)	68.3 (37–88)	<0.0001
Sex					
Male	70 (63%)	11 (85%)	31 (63%)	35 (60%)	0.252
Female	41 (37%)	2 (15%)	18 (37%)	23 (40%)	
Malignancy (%)					
Melanoma	29 (26%)	9 (69%)	8 (16%)	16 (28%)	<0.0001
NSCLC	47 (42%)	2 (15%)	12 (24%)	35 (60%)	<0.0001
RCC	35 (32%)	2 (15%)	29 (59%)	7 (12%)	<0.0001
Prior chemotherapy (%)	62 (56%)	5 (38%)	38 (78%)	24 (41%)	0.0004
Concomitant chemotherapy (%)	25 (23%)	6 (46%)	11 (22%)	14 (24%)	0.08
Steroid use (%)	35 (32%)	7 (54%)	14 (29%)	18 (31%)	0.21
Number of doses (range)	6.1 (2–22)	5.1 (2–17)	7.1 (2–22)	5.6 (2–17)	0.127
Duration of therapy (days) (range)	120 (11–1155)	157 (21–1155)	100 (11–308)	107 (21–350)	0.001

^a^ Note: Seven patients received combination immunotherapy.

### 3.2 Type of infection

**FIGURE 1:**
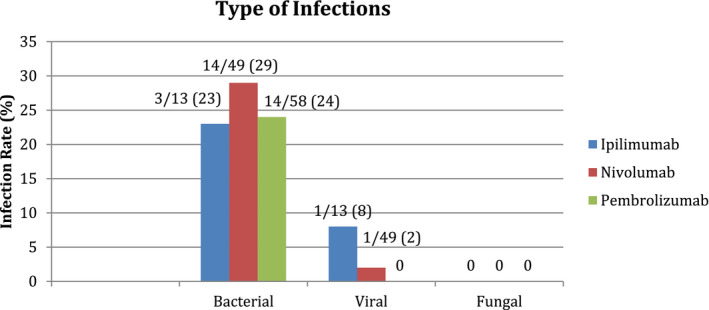
Infection by type

**FIGURE 2:**
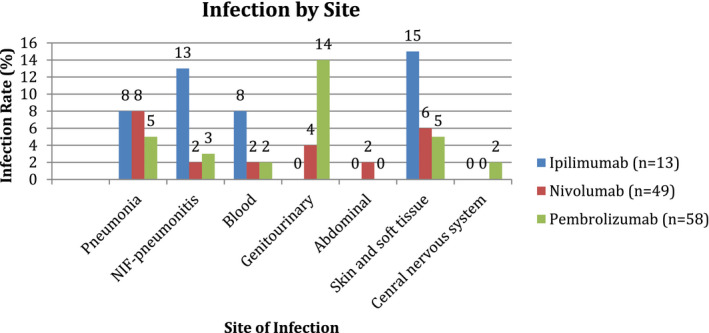
Infection by site. NIF‐pneumonitis, noninfectious pneumonitis

Of the 111 patients included in our study, suspected or confirmed bacterial infections occurred in 24% (27/111) with 8% (9/111) confirmed bacterial cultures. Overall bacterial infections occurred in 23% (3/13) of patients receiving ipilimumab, 29% (14/49) nivolumab, and 24% (14/58) pembrolizumab (Figure 1) with four patients counted twice because they received combination ICI at the time of infection. The ipilimumab group had the highest rate of skin and soft tissue infections (15%, 5/13), whereas genitourinary tract infections (14%, 8/58) were most common in the pembrolizumab group. Bacteremia (*n* = 4), intraabdominal (*n* = 1), and central nervous system (*n* = 1) infections were uncommon in all ICI groups (Figure 2). The median onset of infection from starting ICI was 45.5 days (range 4–319). One patient with bacterial infection had coinfection with *rhinovirus*/*enterovirus*. Otherwise, no additional viral infections were identified. No fungal or parasitic infections were identified. Pneumonia occurred in seven patients with one patient counted twice because of combination ipilimumab and nivolumab therapy with infection rates of 8% (1/13), 8% (4/49), and 5% (3/58) among ipilimumab, nivolumab, and pembrolizumab groups, respectively, with 4/7 patients requiring hospitalization due to pneumonia. Sputum cultures were obtained in 2/7 patients in the pneumonia group and the majority were treated empirically. NIP occurred in five patients with one patient counted twice because of combination ipilimumab and nivolumab therapy. NIP rates were 13% (2/13) receiving ipilimumab compared with 2% (1/49) and 3% (2/58) among the nivolumab and pembrolizumab groups (Figure 2) with 4/5 patients requiring hospitalization due to NIP. Bronchoalveolar lavage fluid was obtained in 2/5 patients in the NIP group.

### 3.3 Microbiology

Among 111 patients, 15 bacterial organisms and 1 viral infection were isolated. Four bloodstream infections: 2 *Bacillus cereus*, 1 *coagulase*‐*negative Staphylococcus*, and 1 *Staphylococcus aureus*. Out of the 22 patients who presented with genitourinary tract infections, 12 had positive cultures with the majority of isolates as extended‐spectrum beta lactamase *Escherichia coli* (*n* = 4) and *Klebsiella pneumoniae* (*n* = 4) (Table 2). Five of the eight patients with skin and soft tissue infections had a pathogen isolated: 1 *Actinomyces radingae*, 1 *Enterobacter cloacae*, 1 *Enterococcus faecalis*, 2 methicillin‐resistant *Staphylococcus aureus*. Only one patient had a viral infection, with a bronchoalveolar lavage positive for *rhinovirus*/*enterovirus* by polymerase chain reaction (PCR—Biofire 1.7 panel). No organisms were identified from patients who had suspected abdominal or central nervous system infection.

**TABLE 2:** Site of infection and organisms identified^a^

	Number of cases
Pneumonia	7
No organisms isolated	7
Noninfectious Pneumonitis^b^	5
Enterovirus/rhinovirus	1
No organisms isolated	4
Bloodstream	4
*Bacillus cereus*	2
*Coagulase‐negative Staphylococcus*	1
*Staphylococcus aureus*	1
Genitourinary	22
*Citrobacter freundii*	1
*Escherichia coli*	1
*Extended spectrum beta‐lactamase Escherichia coli*	4
*Klebsiella pneumoniae*	4
*Streptococcus viridans*	1
*Streptococcus bovis*	1
No organisms isolated	10
Intraabdominal^c^	1
No organisms isolated	
Skin and Soft Tissue	8
*Actinomyces radingae*	1
*Enterobacter cloacae*	1
*Enterococcus faecalis*	1
Methicillin‐resistant *Staphylococcus aureus*	2
No organisms isolated	3
Central Nervous System^d^	1
No organism identified	1

^a^ Patients may have more than one infection.

^b^ Noninfectious pneumonitis was defined as diffuse infiltrates in both lung fields deemed to be related to ICI and work‐up for infection was unrevealing.

^c^ Abdominal: 60 yo M with RCC presented with lower abdominal pain with CT scan consistent with acute diverticulitis treated with IV piperacillin/tazobactam.

^d^ Central nervous system: 72 yo F with NSCLC with metastasis to the brain presenting with confusion, hydrocephalus on CT, and positive Quantiferon, CSF cultures negative, unclear if CSF findings were due to carcinoma or infection.

### 3.4 Serious infections

Patients were classified as having a serious infection if they required hospitalization and/or intravenous antibiotics any time from the initiation of ICI during the study period (Table 3). Serious infections occurred in 16 of 111 patients (14%) and predominantly in patients with nonsmall‐cell lung cancer (*n* = 10). Patients treated with Ipilimumab had the highest number of serious infections (31%; *p *= 0.18) but not statistically significant. Among seven patients who received combination therapy of ipilimumab with nivolumab or pembrolizumab 2 developed a serious infection. Serious infections occurred early on with ICI, 4 versus 6 doses (*p *= 0.036). Among the uncomplicated group (*n* = 95), 23% (22/95) had infections treated with oral antibiotics that did not require hospital admission, whereas 77% (73/95) had no evidence of infection (Table 4).

**TABLE 3:** Serious infections^a^

Characteristics	Total (*n* = 111) (%)	*p*‐value
Total	16 (14%)	
Malignancy		0.139
Melanoma (*n* = 29)	4 (14%)	
NSCLC (*n* = 47)	10 (21%)	
RCC (*n* = 35)	2 (6%)	
Checkpoint inhibitor		0.18
Ipilimumab (*n* = 13)	4 (31%)	
Nivolumab (*n* = 49)	5 (10%)	
Pembrolizumab (*n* = 58)	9 (16%)	
Combination therapy		
I+N (*n* = 6)	2 (33%)	
I+P (*n* = 1)	0 (0%)	

^a^ Serious infection defined as requiring hospitalization and/or intravenous antimicrobials occurring any time from initiation of immune checkpoint inhibitor until December 1, 2017. I+N, ipilimumab and nivolumab; I+P, ipilimumab and pembrolizumab; NSCLC, nonsmall‐cell lung cancer; RCC, Renal Cell Carcinoma.

**TABLE 4:** Risk factors for serious infections

	Serious infection (*n* = 16)	Uncomplicated Group (*n* = 95)	*p* value
Age (mean)	65.8	65	0.81
Male (%)	13 (81.3%)	64 (67.4%)	0.265
Prior chemotherapy (%)	7 (43.8%)	60 (63.2%)	0.142
Concurrent chemotherapy (%)	4 (25%)	21 (22%)	0.798
Steroid use (%)	12 (75%)	27 (28.4%)	0.0003
Number of doses (mean)	4.1	6.5	0.036
Duration of therapy (days) (mean)	76.4	128	0.161

### 3.5 Risk factors for serious infections

Age and gender distribution were similar between patients who had serious infections and nonserious infections (Table 4). In addition, prior chemotherapy or the duration of ICI therapy was not associated with an increased risk of serious infections. Patients who did not meet the criteria for serious infection received more doses of ICIs compared to those with serious infections (6.5 vs. 4.1 doses, *p *= 0.036). Corticosteroid use was higher in the group that had serious infections, 75% (12/16) versus 28.4% (27/95), *p* = 0.0003. No patients received other immunosuppressive agents such as temozolomide or infliximab.

## 4 DISCUSSION

This study aimed to describe the real‐world experience of serious infectious complications from ICIs in melanoma, renal cell carcinoma, and nonsmall‐cell lung cancer patients. Limited data exist in exploring infectious complications of ICI use for various indications. The overall rate of serious infections in our study was 14% (16/111) which is higher compared with a study in melanoma patients which reported an infection rate of 7.3% using a similar definition for suspected serious infection.^1^ Our findings may be higher than De Castillo et al. since we included other cancer types (melanoma, NSCLC, and genitourinary cancers). We also found that using a broad definition of suspected serious infection (e.g., hospitalization requiring IV antibiotics) makes it difficult to separate infectious versus noninfectious complications leading to a higher reported rate. For example, in our study, 5 of 16 met the definition for suspected serious infection but were later found to have NIP (*n* = 3) or sepsis of unknown etiology (*n* = 2). Other reports in the literature that used a broader definition of infection to include outpatient treatment and hospitalization may have contributed to their infection rate that is higher than our study (18–19%).^1^, ^2^, ^3^ The median time to onset of serious infection from initiation of ICI in our cohort was 45.5 days (range 4–319) confirming anecdotes in the literature.^3^ Our results along with reported literature highlight the importance of close monitoring for infection during the first few weeks of ICI therapy. Clinicians should also be aware of the variability in the clinical presentation and the time to onset of infection thus the vigilance to monitor for signs and symptoms of infection well beyond the time of ICI therapy initiation. We observed that those who received fewer ICI doses had serious infections. We hypothesize that serious infections requiring hospital admission may have led to an interruption in ICI therapy compared with those who received more doses during the study period.

Bacterial infections were the predominant cause for infection after ICI. The genitourinary tract was the most common site affected followed by pneumonia and skin and soft tissue infections, which is consistent with published clinical trials.^9^ Only two patients had respiratory sample microbiologic testing with negative results with all patients empirically treated for pneumonia with antibiotics. NIP attributable to ICI occurred in 5% of our total population consistent with reported incidence rates in the literature.^10^ One patient with NIP had a concurrent viral shedding/infection with enterovirus/rhinovirus. None of the patients in our cohort had a fungal infection based on EORTC/MSGERC criteria in contrast to other reports in the literature.^1^, ^2^, ^8^ While we found no association with the specific ICI or type of cancer, corticosteroid use was a significant risk factor that was associated with serious infections. Previous reports describe corticosteroid and infliximab use as risk factors for serious infection; however, none of our patients received infliximab so we are unable to determine if infliximab was also a contributing factor.^1^ Del Castillo et al. observed that patients receiving the combination of nivolumab and ipilimumab were more likely to develop serious infections due to the possibility of a higher incidence of irAEs in patients with melanoma.^8^ In our study, we noted that two out of six patients receiving combination with nivolumab and ipilimumab for melanoma and nonsmall‐cell lung cancer developed serious infections. Our number of serious infections in the combination group was too small to make a definitive statement on the effect of combination therapy on the development of serious infection.

There are important limitations to our retrospective, single‐center experience. Definition of pneumonia and ICI‐induced pneumonitis can be tricky as both can present with varying radiographic findings especially with a lack of uniform approach to diagnostic evaluation of pulmonary symptoms and signs. Lack of microbiologic evaluation from the lower respiratory tract could underestimate the true incidence of infection as a cause of pulmonary infiltrates and pulmonary iRAE can copresent with an infection. Even though every imaging study (chest X‐ray and CT scan of chest) was reviewed by a physician and correlated with clinical description, it does not entirely eliminate the above‐stated concern. Also, our sample size was relatively small, and the duration of this study was relatively short compared with a previous study that reviewed 740 patients with melanoma over 4 years. Last, as ICI is commonly given in an outpatient setting, it is likely that rigorous workup for the etiologic cause of infection may not occur consistently. This often leads to empiric treatment with antimicrobials with the absence or lack of microbiologic data, and it becomes difficult to determine if symptoms were due to infection, disease, or iRAEs. Therefore, we chose to analyze the incidence of serious infections rather than all infection severity types to maintain a consistent population that underwent appropriate infectious disease workup.

## 5 CONCLUSION

In summary, we observed a serious infection rate of 14% in patients with melanoma, renal cell carcinoma, and nonsmall‐cell lung cancer treated with ICIs. In this study, the number of ICI doses administered and the use of steroids to treat patients with iRAEs were significantly associated with serious infections. Further studies are needed to differentiate between the incidence of iRAEs and the infection.

## CONFLICTS OF INTEREST

We know of no conflicts of interest associated with this publication, and there has been no financial support for this work that could have influenced its outcome.
